# Implantation of Human Teeth

**Published:** 1888-09

**Authors:** Edward C. Kirk

**Affiliations:** Philadelphia, Pa.


### Independent Practitioner.

Vol. IX. September, 1888. No. 9.

Note.—No paper published or to be published in another journal will be accepted for this department. All papers must be in the hands of the Editor before the first day of the month preceding that in which they are expected to appear. Extra copies will be furnished to each contributor of an accepted original article, and reprints, in pamphlet form, may be had at the cost of the paper, press-work and binding, if ordered when the manuscript is forwarded. The Editor and Publishers are not responsible for the opinions expressed by contributors. The journal is issued promptly, on the first day of each month.

### Original Communications.

### IMPLANTATION OF HUMAN TEETH.

(Younger's Operation.)

BY EDWARD C. KIRK, D. D. S., PHILADELPHIA, PA.

Read before the Pennsylvania State Society, June 6, 1888.

The operation of implanting natural teeth in artificial sockets formed in the alveolar ridge is still in the experimental stage, but enough has been done to throw some light on the questions of its expediency, utility, and possible permanency. The operation as devised and performed by Dr. W. J. Younger differs in its essential details from the somewhat analogous operations of transplantation and replantation by virtue of its distinguishing characteristic, viz: the making of the socket by operation. To what extent the artificial socket is a factor in the production of a successful result cannot as yet be definitely stated; it is believed, however, by those who look upon the operation with favor, that the making of a socket artificially, contributes to its success, and possesses a decided advantage over the planting of teeth in natural sockets, which are, or have been, the seat of disease.

Younger's method of performing the operation is to make a linear incision through the gum and soft tissues down to the alveolar ridge, then with a sharp chisel carefully dissect the periosteum from the bone on either side. The soft tissues form a flap, which, when the tooth is finally inserted, encircles it, and helps to hold it in position. The socket is formed in the bone, first by means of a graded set of trephines, which are then followed by spirally cut burs or reamers adapted in size to the dimensions of the root to be inserted. The tooth is prepared for introduction by removing the pulp through the apical foramen, which is sufficiently enlarged for the purpose, and filling the pulp-chamber and canal with gutta-percha, and the apex with gold, which must be smoothly finished. The tooth is then subjected to the action of a mercuric bichloride solution, 1 to 1,000, for fifteen or twenty minutes, at a temperature of 108 or 110 deg. F., which completely sterilizes it. The socket is carefully washed out with warm water to remove all debris, and then with pledgets of cotton, saturated with the bichloride solution. The tooth is then inserted, and if necessary, retained by ligatures. Union generally takes place within a few days, and at the expiration of a week, the ligatures may be removed. The rapidity and manner of the process of repair varies greatly in different cases, and so far as a limited clinical experience can determine, seems to be dependent upon two sets of causes. First, the care as to certain details and manner of performing the operation; and second, the patient's condition of health.

The first factor, viz.: the manner of performing the operation, exerts a marked and decided influence upon the healing process, and probably upon the final result. It must be borne in mind that a body foreign to the tissues is introduced; foreign, because devoid of vitality, and which may become an irritant of sufficient intensity to defeat the object in view, by inducing such a high state of inflammation as to cause suppuration, if not sloughing, or at least failure to unite. Our object is, therefore, to so control the inflammatory process which the irritation of this foreign body has set up, that it shall not at any time exceed the degree necessary for repair of the wound, and a complete encapsulation of the root.

The method pursued by the writer, and which differs in some of its details from that of Dr. Younger, is briefly as follows :— An incision is made through the gum with a small "tenotome," and the periosteum reflected from the bone in the same manner as that followed by Younger; the process is then entered, and a cut made through the bone the desired depth, with a wide and measured spear-pointed drill, mounted in the Bonwill engine, having a driving wheel of the largest diameter. The drill is flat, about three thirty-seconds of an inch in diameter, and sharpened to a keen edge. Having a speed of from 10,000 to 30,000 revolutions at command, the cut is made instantly and almost painlessly. The drill cut is followed by coarse-cut reamers, of which I use two sizes, the larger having five, and the smaller, four very sharp cutting leaflets, winding spirally about the shank. With these instruments the socket is formed. I was led to the use of these coarse-cut instruments by finding that those with shallower leaflets became clogged and heated while cutting the bone tissue, thus needlessly prolonging the operation, and greatly increasing the pain. I find that the coarse-cut reamers obviate these objections entirely, and by running the engine at a high speed, cut very smoothly, and with but little jar, on the same principle that the coarse teeth of a rapidly run circular saw will cut a smooth surface on a board. The socket should now be carefully washed to remove all debris left by the burs, as the smallest spicula of bone or other foreign matter will increase the amount of irritation and subsequent inflammation, and add greatly to the discomfort of the patient, while at the same time retarding the process of repair. The preparation of the tooth is the same as that pursued by Younger so far as the filling of the root is concerned. The sterilization is effected in a special apparatus devised by myself for the purpose. It consists of a rectangular copper box, nickel plated, with a hinged lid perforated with three holes for the reception of three glass vessels, in one of which may be placed mercuric bichloride solution, 1 to 1,000, in which the tooth is placed for about twenty minutes before it is inserted; in another, a somewhat stronger solution, about 1 to 500 for instruments, and in the third, ordinary distilled water.

When forming the socket, great care must be taken to leave sufficient thickness of gum tissue on the labial or buccal aspect of the socket, and to avoid puncturing it at this point. If only a very thin covering is left for the root on its buccal or labial aspect, the distention of the tissue on that side of the socket, caused by the introduction of the root, is apt to strangulate the circulation of the part, and result in partial or complete absorption of that portion of the socket wall. Should the outer wall of the socket be accidentally punctured, failure to unite at that point will most surely follow, and leave a denuded patch of the root exposed to view, which not only presents an unsightly appearance, but to a considerable extent endangers the permanency of the result.

My first case of implantation was performed November 30, 1886. The patient, a lady about thirty years of age, had lost the first superior right bicuspid ten years before. A suitable tooth was treated and implanted after the manner of Younger; union took place by first intention, and at the expiration of three weeks, the tooth was in no way distinguishable from the other teeth. A noticable feature of this case, and one which has occurred in a majority of the cases of the writer, is that on the third or fourth day after the operation, a considerable exudation of plasma took place under the gum margin, in the tissues around the neck of the implanted tooth, forming a distinct ring and giving a thickened appearance to the gum. This thickened ring, which, during the first week or ten days, was quite soft to the touch gradually became harder, until finally it became rigid and unyielding, and presented to the touch all the physical characteristics of a bony callous. Upon percussing the tooth with a steel instrument, a peculiar and distinct resonance, totally different from the rest of the other teeth, was evolved, the impression produced being that of a bony ankylosis or direct union with the ridge without any intervening membrane. That this was the case is still further indicated by the fact that such teeth are perfectly devoid of that slight mobility common to normal teeth.

The condition just described has, with two exceptions, been typical of thirty-three teeth implanted by the writer up to the present time, and so far as a close clinical observation of each case can determine, seems to be the normal process of repair of the tissues in implantation.

When we consider the process by which a wound of the tissues involved in the operation of implantation is repaired under normal conditions, and in which we do not have to deal with the tooth as a factor; the rationale of the method is easily understood. Immediately following the incision, an exudation of leucocytes or so-called plasma cells takes place; these become organized and develop into connective tissue with its capillary blood-vessel system, following which, in bone, a deposit of calcific material takes place, and ossification ensues through the agency of the osteoblastic cells which belong to the connective tissue group.

Under unfavorable conditions, that is, where from any cause the irritation is sufficiently great to set up and maintain for a time a high state of inflammation, giant cells or osteoclasts are developed, and absorption takes place through their agency until the cause of the irritation is completely removed. It is possible for the inflammatory process to abate and a normal condition of the tissues result, whereby the reparative process first alluded to will follow even after a considerable degree of absorption has taken place.

In view of the foregoing considerations, therefore, it is of the greatest importance that in all cases of implantation, the inflammation caused by the operation, and to a certain degree maintained by the presence of the tooth, which acts, or may act, as an irritant, should be kept within the narrowest possible limits to insure the very best result in each case; the means for accomplishing this may be briefly summed up as follows:

First—The careful selection of a tooth, nicely adapted to the case, and having its pericemental membrane intact, and its proper preparation, by removing the pulp and filling the chamber and canal. The latter may be accomplished by enlarging the apical foramen or cutting through the palatine surface, but if by the former, special care must be exercised in finishing the final gold cap, so that it shall be absolutely free from roughness or projecting points that may become the cause of irritation.

Second—The formation of the socket with sterilized instruments, as carefully and as rapidly as is consistent with thoroughness. Following this with the complete removal of every particle of debris, and the sterilization of the socket and tissues surrounding it.

Third—The absolute sterilization of the tooth as before described, and the maintenance of antiseptic conditions around it, and throughout the oral cavity, by the use of phenol sodique or its equivalent, until repair has been completed.

The last fifteen operations which I have performed have been done under the influence of cocaine as a local anæsthetic. The action of the drug in these cases has been so satisfactory that in no instance where it has been given time to act, has any pain whatever been felt. The method which I pursue is to inject into the gum tissue in a line with the proposed socket, from a half minim to a minim of a fifty per cent solution of cocaine hydrochlorate. The full anæsthetic effect is reached in about ten minutes, when the tissues can be cut and the socket formed entirely without pain. The reason for using so strong a solution is that it requires about a quarter of a grain of the cocaine salt to produce a sufficiently profound effect. The tissues being rigid and unyielding, it is difficult to inject a larger quantity of the solution which would be required if a more dilute solution were used.

I have had no unpleasant results, either local or general, from the use of the drug in this manner, nor does it seem to have any effect whatever upon the process of repair. It renders the operation absolutely devoid of pain, the only disagreeable feature complained of by the patient being the slight painful sensation produced by the introduction of the hypodermic needle. This, however, is but momentary.

Whether this operation will become one of the recognized procedures in operative dentistry or not, will depend upon the measure of success attained, and just what constitutes a successful operation of this sort is an open question; that failures do occur from absorption of the roots of the planted tooth we are all aware. This loss of the tooth may occur at variable periods of time, ranging from a few months to several years, but the same thing, that is, absorption of the root, occurs in normal teeth, and is frequently due to causes which we are unable at present to fathom. Compared with many of the other operations that are performed for the conservation of the natural teeth, the record would seem to afford a conclusion favorable to implantation.

I have, out of thirty-three operations which I have done, lost three teeth, two of which were in the same mouth, that of a young man suffering from secondary syphilis, of which I was not aware at the time of operating. This case I reported in the *Dental Cosmos.*

The other case in which the tooth was lost was that of an apparently healthy young man of fine physical appearance, for whom I implanted a left superior cuspid in October last. He reported to me last week that "while biting a hard crust of bread, he felt the tooth suddenly break with a noise" which he described as "like a pistol shot." On examination it was shown to have been largely attacked by osteoclasts, which had formed deep, bay-like excavations in the cementum and dentine, and which had weakened the tooth to such an extent that it had sustained a transverse fracture, at about the middle point of its length, from the force incident to masticating a rather hard bread-crust.

It is, perhaps, worthy of note that in both instances where the teeth were lost, absorption had taken place about six months after implantation.

The following extract from a note from Dr. H. C. Herring, of Concord, N. C., for whom I implanted a superior lateral incisor, so well represents the case from the standpoint of the patient, that I take the liberty of reading it:

"There was the slightest pain during the operation. No subsequent soreness until the afternoon of the 20th. It was nothing similar to periodontitis; but a soreness analogous to an insect bite, without any swelling or visible inflammation. It had entirely subsided by morning. The eighth day I removed the ligatures, as they were causing trouble to the gums of the adjoining teeth. Since then it has given neither annoyance nor trouble. Upon the other hand it has always felt perfectly comfortable. It is now as firm as the others, and while I do not subject it to any hard usage, yet I am sure it can do the work of the others. It has turned about two shades darker, but is still about one shade too light. As compared to a partial plate, I do not regard the operation as the "lesser of the two evils," but a real boon, a God-send. And he who will throw away his partial piece or tear out an anterior bridge and avail himself of this, well! surgical enigma, will rise up and call it blessed. I am so delighted; if needs be, I would willingly submit to the operation every six months."

What will be the final status of Younger's operation? is a question that can only be decided by the crucial test of time and experience. All innovations have, throughout the world's history, been met by opposition and adverse criticism, which have served the purpose of stimulating their champions to greater endeavor or resulted in their defeat, and Dr. Younger's operation has not been an exception; it is still in the probationary stage, but at the same time gives much promise of occupying a high place in the catalogue of future dental operations. It has been called "unsurgical," because it is unique; but the highest ideal of surgery is or should be conservative, and it reaches its fullest expression when we save an injured member or restore a lost one; the amputation of a limb and the extraction of a tooth are practical confessions of surgical defeat. It has been fully demonstrated by Younger, and those who have followed him, that without regard to any accepted theory of the past, teeth may be planted after his method and become firmly fastened in the alveolus by some means sufficiently strong to perfectly fulfill the requirements of normal teeth, at least for a time.

The discovery that Dr. Younger made and had the courage to promulgate should entitle him to the honor and thanks of every man in the dental profession who has the advancement of his calling at heart. He has done all that any man can up to date in the matter of demonstrating, by his operations in this line, what was heretofore unknown and even believed to be impossible. That it shall prove to be a final success is desired by every one, as no method of restoring a broken dental arch to usefulness and symmetry by artificial substitution bears favorable comparison with it; and if time shall show that implanted teeth can be retained but two or three years as a maximum limit, I should not hesitate to recommend and practice it after the satisfactory experience I have had in its performance, because, by the methods which I have detailed, it is nearly painless, the ordinary operations of filling far exceeding it in this respect, and I also believe it to be free from danger.

